# Long-Term Performance of Feldspathic and Lithium Disilicate Restorations in Pediatric Anterior Dental Trauma

**DOI:** 10.3390/children12081019

**Published:** 2025-08-01

**Authors:** Sorin Gheorghe Mihali, Șerban Talpoș, Dan Loloș, Bogdan Antonio Loloș, Andreea Raissa Hojda, Loredana Mitariu

**Affiliations:** 1 Department of Prosthodontics, Faculty of Dentistry, “Vasile Goldiș” Western University of Arad, 94 Revoluției Blvd., 310025 Arad, Romania; mihali.sorin@uvvg.ro; 2 Discipline of Oral and Maxillo-Facial Surgery, Faculty of Dental Medicine, “Victor Babeș” University of Medicine and Pharmacy Timișoara, Revoluției Boulevard 9, 300041 Timișoara, Romania; 3 Faculty of Dental Medicine, “Victor Babeș” University of Medicine and Pharmacy Timișoara, Eftimie Murgu Square No. 2, 300041 Timișoara, Romania; lolosdan@umft.ro; 4 Faculty of Medicine, “Vasile Goldiș” Western University of Arad, 94 Revoluției Blvd., 310025 Arad, Romania; lolosantonio@yahoo.com; 5 Faculty of Medicine, “Victor Babeș” University of Medicine and Pharmacy Timișoara, Eftimie Murgu Square No. 2, 300041 Timișoara, Romania; andreea.hojda@student.umft.ro; 6 Department IV of Dental Medicine and Nursing, Faculty of Dentistry, University of Sibiu “Lucian Blaga” (ULBS), Lucian Blaga 2A, 550024 Sibiu, Romania; loredana.mitariu@ulbsibiu.ro

**Keywords:** pediatric dental trauma, feldspathic ceramic, lithium disilicate, anterior restorations, ceramic crowns, veneers, adolescent dentistry, minimally invasive technique

## Abstract

**Background/Objectives**: Anterior dental trauma in adolescents presents complex restorative challenges due to ongoing craniofacial development and high aesthetic expectations. This study evaluated the long-term clinical performance of feldspathic ceramic veneers and lithium disilicate crowns used in the anterior region following dental trauma in adolescents. **Methods**: A total of 209 restorations were placed in 85 adolescents (50 females, 35 males), aged 11.1–17.9 years (mean age: 15.1 years). Of these, 144 were lithium disilicate crowns, and 65 were feldspathic ceramic veneers. All restorations were fabricated using minimally invasive protocols and followed up for periods ranging from 3 to 60 months. Outcomes were assessed based on standardized clinical criteria for success and failure. **Results**: Lithium disilicate crowns exhibited superior long-term performance, with the majority of failures occurring in feldspathic veneers (*p* < 0.001), primarily due to chipping or structural fracture. Age and gender had no statistically significant influence on failure rates. **Conclusions**: Both feldspathic and lithium disilicate ceramic restorations represent viable treatment options for anterior dental trauma in adolescents. However, lithium disilicate demonstrates greater mechanical reliability, particularly in teeth with significant hard tissue loss. These results support the use of durable ceramic materials in adolescent restorative protocols involving dental trauma.

## 1. Introduction

Dental trauma in children, particularly in the anterior region, presents a significant challenge from both aesthetic and functional perspectives. Such injuries are common, often resulting from accidents during playground activities, sports, or educational environments, and affect approximately 15–20% of children globally [1,2]. The anterior region is most frequently impacted, with the prevalence notably higher among children participating in vigorous physical activities. These traumatic events can have profound consequences on a child’s oral health, self-esteem, and social development, frequently leading to long-term effects on their quality of life and interpersonal relationships [3,4].

In adolescent patients, craniofacial growth, increased pulp chamber volume, and dynamic occlusal development lead to different functional and biological conditions compared to adults. Moreover, adolescents often have heightened aesthetic demands, and once the superior optical properties of feldspathic ceramics are understood, their preference for highly natural restorations increases [3]. At the same time, masticatory forces in this age group are lower than in adults, which initially favors more delicate materials, yet the need for long-term durability in structurally compromised teeth may justify the use of lithium disilicate. These specific biomechanical and aesthetic considerations highlight the necessity of minimally invasive yet age-appropriate restorative strategies and justify a separate evaluation of feldspathic and lithium disilicate restorations in the pediatric adolescent population.

Restoring the function and appearance of traumatized teeth in adolescents presents distinct challenges. Treatment planning must take into account the continuous development of the child’s oral anatomy, including the dentition and skeletal structure. Restorative interventions must be designed to accommodate potential changes in tooth and jaw growth, ensuring that the restorations remain functional and aesthetically integrated as the child matures. This dynamic aspect of pediatric treatment complicates the execution and long-term success of restorative procedures, particularly in the case of traumatic injuries to the anterior teeth [5,6].

Feldspathic ceramics are especially well-suited for pediatric anterior restorations due to their ability to replicate the natural morphology and translucency of enamel. This is crucial for mimicking the unique growth lobes and surface characteristics of developing anterior teeth, essential for achieving a natural and aesthetically pleasing result. Additionally, feldspathic ceramics allow dental technicians to replicate the adjacent, unaffected teeth with a high degree of precision, which is vital when restoring the highly visible anterior region [7,8].

In this study, we have chosen feldspathic ceramic and layered lithium disilicate (e.max) restorations for their complementary aesthetic properties and their capacity to provide durable, long-lasting solutions in these challenging clinical cases. While feldspathic ceramics excel at replicating enamel’s translucency, lithium disilicate offers superior strength and fracture resistance, making it ideal for cases requiring greater durability [9,10]. These materials are widely recognized for their ability to meet the aesthetic and functional demands of anterior trauma restoration in adolescents [11,12].

The management of pediatric dental trauma typically involves a multidisciplinary approach, incorporating both pediatric dentists and prosthodontists. Although these cases are rarely referred directly to prosthodontists, they are often managed collaboratively to ensure comprehensive care. Such a combined effort allows for the development of tailored treatment plans that not only address immediate functional and aesthetic needs but also anticipate long-term considerations, such as future changes in occlusion and maxillofacial growth. By working together, specialists can offer a holistic solution that supports both the present and future dental development of the child [13,14].

A major challenge in restorative dentistry for children is the limited availability of data on minimally invasive techniques using materials like feldspathic ceramics and lithium disilicate, particularly in cases of anterior trauma. Minimally invasive treatments focus on preserving as much natural tooth structure as possible. However, in cases involving substantial hard tissue loss, more conservative options, such as direct composite restorations, may not be feasible. In such instances, feldspathic veneers offer excellent aesthetic results, effectively replicating the natural morphology and translucency of anterior teeth. When significant dentin loss is present, layered lithium disilicate (e.max) restorations provide both aesthetic precision and mechanical strength, offering a durable yet minimally invasive solution for adolescents [15,16].

Compared to feldspathic ceramics, layered lithium disilicate e.max restorations offer significantly higher fracture resistance, with flexural strength values reported between 400 and 500 MPa, in contrast to feldspathic ceramics, which exhibit a strength of approximately 100–120 MPa [17,18,19]. This makes layered lithium disilicate a preferred option in cases requiring greater durability, particularly for restorations in regions exposed to substantial occlusal forces [11,20].

Despite their promising potential, the long-term performance of feldspathic ceramics and lithium disilicate restorations in pediatric populations remains underexplored. Few studies have specifically examined the survival rates and clinical behavior of these materials in pediatric dental trauma, particularly in the anterior region. This gap in the literature underscores the need for further research to establish the long-term viability of these materials in young patients and to evaluate their effectiveness in minimally invasive restorative procedures [21,22].

The present study aims to address this gap by examining the clinical outcomes of feldspathic and lithium disilicate restorations in pediatric anterior trauma cases. By focusing on the functional and aesthetic results of these materials in young patients, we hope to provide valuable insights into their long-term effectiveness and inform future restorative approaches for pediatric dental trauma [23,24,25,26].

Therefore, the objective of this study was to evaluate and compare the long-term clinical performance of feldspathic ceramic veneers and layered lithium disilicate crowns in anterior restorations among adolescents who experienced dental trauma. Specific outcomes assessed included restoration longevity, failure rates, and distribution of failure causes, with additional analysis of demographic and clinical variables. Through this investigation, we aimed to identify the most suitable restorative approach in terms of durability and esthetic integration, offering evidence-based recommendations for managing anterior dental trauma in adolescents using minimally invasive ceramic techniques.

## 2. Materials and Methods

This prospective clinical study evaluated the long-term performance of feldspathic veneers and lithium disilicate crowns in anterior dental trauma cases among adolescents. All procedures were performed at the private dental clinic Dental Concept by Dr. Mihali (Timișoara, Romania), following standardized therapeutic protocols, and all restorations were carried out by the same clinician to ensure consistency in technique and clinical execution. The study adhered to the ethical principles outlined in the Declaration of Helsinki and was approved by the Ethics Committee of the “Victor Babeș” University of Medicine and Pharmacy in Timișoara, Romania (Approval Nr. 92/02.05.2022, revised 2025/3). Written informed consent was obtained from the parents or legal guardians of all participants. Patient privacy, safety, and autonomy were fully respected throughout the study period.

The study enrolled 85 patients, including 50 girls (mean age: 182 months, approximately 15 years and 2 months) and 35 boys (mean age: 180 months, approximately 15 years), with ages ranging from 133 to 215 months (11 years and 1 month to 17 years and 11 months). A total of 209 ceramic restorations were placed: 65 feldspathic veneers and 144 lithium disilicate crowns; they were all located in the anterior region. The follow-up period, excluding restorations that were classified as failures, ranged from 20 to 77 months.

Restorations were classified as ‘Failure’ when they presented ceramic fracture involving more than one-third of the restoration or recurrent chipping requiring clinical repair or replacement. Minor superficial wear without structural compromise was not considered a failure. In this study, all recorded failures were related to ceramic fracture (*n* = 7) or chipping (*n* = 3) of feldspathic veneers; no debonding, secondary caries, or loss of vitality occurred during the follow-up period.

All participants were adolescents within the pediatric age group, as they were minors under 18 years old at the time of treatment. For clarity, the term “adolescent patients” is used throughout this manuscript to specify this subgroup of the pediatric population.

Participant selection followed clear clinical criteria. Eligible patients had sustained anterior dental trauma involving one or more permanent incisors, with teeth sufficiently erupted and displaying complete or nearly complete root development. A lower age limit of 11 years was established based on the anatomical maturity of anterior teeth required for adhesive procedures. Other inclusion parameters included the absence of active periodontal disease and the presence of favorable local and systemic conditions for healing. Patients presenting with systemic disorders that could interfere with oral healing, or with teeth exhibiting excessive structural loss beyond the scope of minimally invasive ceramic rehabilitation, were excluded.

Each patient underwent an initial clinical and radiographic evaluation to determine the extent of trauma and guide the treatment plan. Periapical radiographs were routinely performed, while CBCT imaging was employed selectively in cases with suspected root fractures, apical lesions, or alveolar involvement. Pulp vitality was assessed using thermal testing, and occlusal parameters were documented both statically and dynamically, including overjet, overbite, and guidance patterns.

Minimally invasive preparation protocols were consistently followed throughout the clinical workflow.

Feldspathic veneers were applied with either no preparation or a conservative facial reduction between 0.1 and 0.5 mm, typically finished with a chamfer margin. In this study, feldspathic veneers were indicated in cases where enamel fractures did not exceed approximately 3 mm of unsupported structure, although borderline situations were included when sufficient peripheral enamel allowed stable adhesion. Gingival soft tissue management was performed using braided retraction cords (Ultrapak, Ultradent Products Inc., South Jordan, UT, USA) in most cases. In clinical situations requiring a more delicate approach, such as subgingival margins or fragile gingival biotypes, a retraction paste (Expasyl, Acteon Group, Mérignac, France) was used. Conventional impressions were obtained using polyvinyl siloxane material (Virtual, Ivoclar Vivadent, Schaan, Liechtenstein), which allowed for high-fidelity detail capture and was compatible with the refractory die fabrication technique. Temporary restorations were fabricated chairside using bis-acrylic resin (Protemp 4, 3M ESPE, Seefeld, Germany) or mock-ups based on the diagnostic wax-up, allowing patients to preview the final esthetic outcome and facilitating clinical adjustments when necessary.

**Figure 1:**
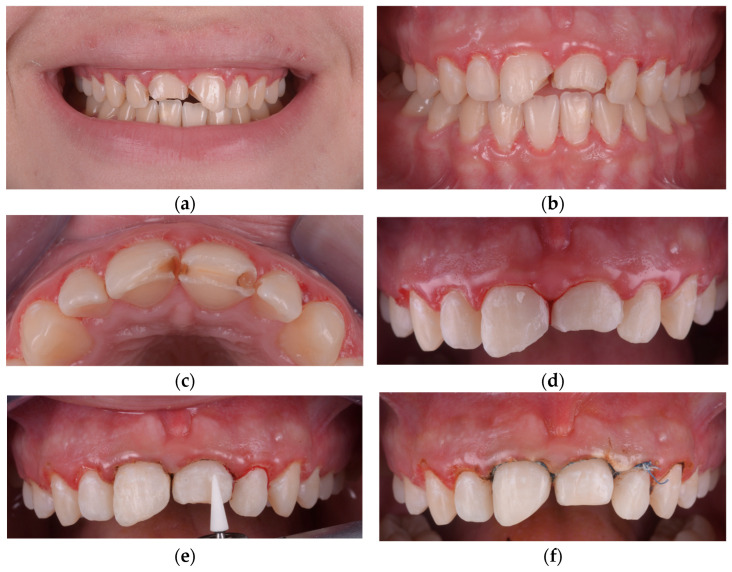
(**a**) Initial extraoral view showing bilateral incisal fractures of the maxillary central incisors. (**b**) Intraoral frontal view highlighting enamel loss. (**c**) Occlusal view of the maxillary arch showing incisal defects and secondary caries on maxillary central incisors. (**d**) Initial phase of labial surface preparation, focused on eliminating sharp edges and creating a single path of insertion. (**e**) Finishing of the preparation margins using a rotary-mounted Arkansas stone (Sitea, Romania) to achieve smooth enamel transitions and prevent marginal stress points. (**f**) Placement of braided retraction cords (Ultrapak, Ultradent Products Inc., South Jordan, UT, USA) to facilitate accurate impression-taking and soft tissue management.

Special attention was given to the finishing of preparation margins, with the removal of sharp edges and concave areas that could interfere with a single path of insertion. Tooth morphology was modified when needed to create favorable contours for optimal veneer seating and esthetic emergence profiles, as seen in Figure 1.

Feldspathic veneers were fabricated using the refractory die technique by the same dental technician to ensure precision, marginal accuracy, and esthetic control.

Prior to final cementation, provisional restorations were removed, and each veneer was evaluated intraorally for proximal contact, marginal integrity, occlusal adaptation, and color matching. The intaglio surface of each veneer was etched with 9.5% hydrofluoric acid (IPS Ceramic Etching Gel, Ivoclar Vivadent, Schaan, Liechtenstein) for 60 s, followed by a 60-second cleaning with 36% orthophosphoric acid (Blue Etch, Cerkamed, Stalowa Wola, Poland). Ultrasonic cleaning was performed in distilled water for 5 min. A silane coupling agent (Monobond Plus, Ivoclar Vivadent, Schaan, Liechtenstein) was then applied for 60 s and gently dried with air to ensure a uniform silane layer.

**Figure 2:**
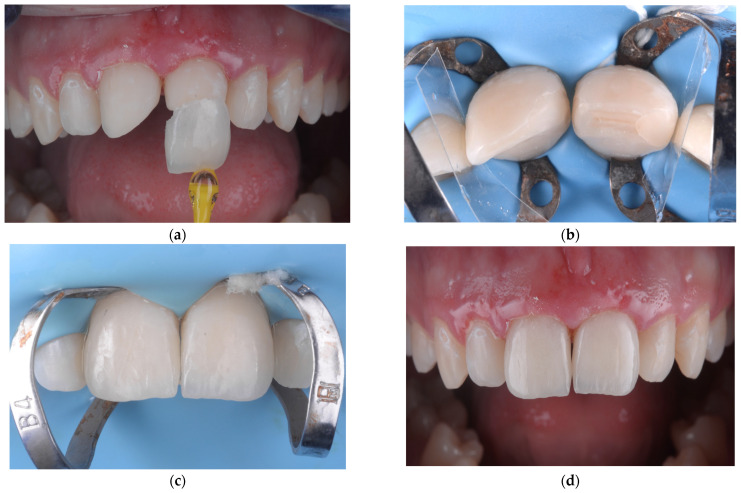
(**a**) Precementation view showing the minimal thickness and natural translucency of the feldspathic ceramic veneer. (**b**) Isolation of maxillary central incisors using a celluloid transparent matrix strip (Andet, Italy) to confine the cementation area and prevent resin excess from spreading onto adjacent teeth. (**c**) Immediate postoperative view following cementation, showing the esthetic integration of the two central incisors. (**d**) Intraoral final aspect of feldspathic ceramic veneers, illustrating the natural appearance and seamless transition with adjacent enamel.

To ensure optimal bonding, the teeth were isolated with rubber dam (Figure 2) and sandblasted with 50 µm aluminum oxide particles (RØNVIG Dental Mfg. A/S, Daugaard, Denmark), then etched with 36% phosphoric acid (Blue Etch, Cerkamed, Stalowa Wola, Poland) for 45 s. After rinsing and drying, a universal adhesive (Adhese Universal VivaPen, Ivoclar Vivadent, Schaan, Liechtenstein) was applied and evenly air-thinned. Cementation was carried out using a dual-cure luting resin cement (Variolink Esthetic LC, Ivoclar Vivadent, Schaan, Liechtenstein), with excess material removed before light curing. Each surface was light-cured for 30 s using a unit delivering approximately 1470 mW/cm^2^, followed by an additional 20-second curing cycle after application of glycerin gel to eliminate the oxygen inhibition layer. Residual cement was meticulously removed using a scaler, dental floss, and a 12D surgical blade. Restoration margins were finished using the OptraFine Diamond Polishing System (Ivoclar Vivadent, Schaan, Liechtenstein) and interproximal polishing strips (Diamond Strips, Komet, Lemgo, Germany). After removal of the rubber dam, final occlusal adjustments were performed in centric relation as well as lateral and protrusive guidance. Final ceramic polishing was achieved using felt wheels and high-performance polishing paste (OptraFine HP Polishing Paste, Ivoclar Vivadent, Schaan, Liechtenstein), providing a smooth, stain-resistant surface.

For lithium disilicate restorations, a similarly conservative protocol was followed, respecting tissue preservation and adhesive principles. Tooth preparation involved incisal and axial reductions between 0.4 and 0.8 mm, sufficient to accommodate the mechanical requirements of the material while minimizing dentin exposure. Soft tissue retraction was primarily achieved with braided cords (Ultrapak, Ultradent Products Inc., South Jordan, UT, USA), while in cases of delicate gingival biotypes or subgingival margins, retraction paste (Expasyl, Acteon Group, Mérignac, France) was selectively employed. A fully digital workflow was implemented for all lithium disilicate crowns. Intraoral scanning was performed using the 3Shape TRIOS system (3Shape, Copenhagen, Denmark), enabling high-resolution acquisition of digital impressions. These were followed by CAD design and heat-press fabrication of the final restorations. All lithium disilicate crowns (IPS e.max Press, Ivoclar Vivadent, Schaan, Liechtenstein) were produced by the same dental technician using the heat-pressing technique, ensuring homogeneity in precision, marginal fit, and optical properties.

Prior to definitive bonding, provisional crowns were removed, and each final crown was assessed intraorally for marginal adaptation, proximal fit, occlusion, and shade integration. The internal surface of each restoration was etched with 5% hydrofluoric acid (IPS Ceramic Etching Gel, Ivoclar Vivadent, Schaan, Liechtenstein) for 20 s, then rinsed thoroughly and cleaned using 36% orthophosphoric acid (Blue Etch, Cerkamed, Stalowa Wola, Poland) for 60 s to remove any residual crystalline debris. The conditioned surface was then treated with a silane coupling agent (Monobond Plus, Ivoclar Vivadent, Schaan, Liechtenstein) applied for 60 s and gently air-dried to form a uniform reactive layer.

Rubber dam isolation was employed to ensure proper field control. The prepared tooth was sandblasted with 50 µm aluminum oxide particles (RØNVIG Dental Mfg. A/S, Daugaard, Denmark) to improve micromechanical retention, then etched with 36% phosphoric acid (Blue Etch, Cerkamed, Stalowa Wola, Poland) for 45 s, rinsed, and air-dried. A universal adhesive (Adhese Universal VivaPen, Ivoclar Vivadent, Schaan, Liechtenstein) was then applied and evenly air-thinned across the surface.

Final cementation was carried out using a dual-cure resin luting composite (Variolink Esthetic DC, Ivoclar Vivadent, Schaan, Liechtenstein). Prior to bonding, the esthetic integration of each restoration was assessed using a try-in procedure with Variolink Esthetic Try-In gel (Ivoclar Vivadent, Schaan, Liechtenstein), allowing for shade evaluation under warm, neutral, and bleach lighting conditions. The proper selection of the lithium disilicate ceramic block was guided by ND (Natural Die) registration of the abutment shade, which enabled accurate matching between the underlying tooth and final restoration. This step was essential to avoid the risk of insufficient masking or unwanted discoloration, as the use of an inadequately opaque ceramic could compromise the final esthetic result.

Once the optimal shade was selected, restorations were seated, and excess cement was gently removed before polymerization. Each surface was light-cured for 30 s with a unit delivering approximately 1470 mW/cm^2^. To eliminate the oxygen inhibition layer and achieve full polymerization at the margins, glycerin gel was applied and followed by an additional 20-second light cure. Residual cement was meticulously removed using hand instruments, scalers, and dental floss. Final polishing of the margins was completed using the OptraFine Diamond Polishing System (Ivoclar Vivadent, Schaan, Liechtenstein) and interproximal strips (Diamond Strips, Komet, Lemgo, Germany), ensuring smooth transitions and high surface gloss. Occlusal adjustments were carried out after removal of the rubber dam, followed by final ceramic polishing using felt wheels and OptraFine HP Polishing Paste (Ivoclar Vivadent, Schaan, Liechtenstein). Patients were monitored through scheduled follow-up appointments. At one month, assessments focused on occlusion, marginal adaptation, and soft tissue response. A six-month recall evaluated functional integrity and esthetic integration. Annual check-ups were conducted thereafter. Each case was categorized as “Success” or “Failure,” with documentation of failure causes and timing when applicable.

Statistical analysis was performed using JASP v0.17.1 software. Quantitative variables were reported as mean ± standard deviation and as median (Quartile1–Quartile3). The Mann–Whitney U test was used to compare two independent groups. Categorical variables were expressed as counts and percentages, with group differences evaluated via the Chi-square test or Fisher’s Exact test where appropriate. Kaplan–Meier survival analysis was employed to assess restoration longevity, and group comparisons were performed using the Log-rank test. Logistic regression was used to identify whether age, sex, restoration type, or follow-up duration were significant predictors of failure. A *p*-value below 0.05 was considered statistically significant.

## 3. Results

The study included 85 adolescents, with a sex distribution of 50 females (58.8%) and 35 males (41.2%). A total of 209 anterior restorations were performed, comprising 65 feldspathic veneers (in 32 patients) and 144 lithium disilicate crowns (in 53 patients), representing 37.6% and 62.4% of the cohort, respectively.

The average patient age was 180.98 months (approximately 15 years), ranging from 133 to 215 months. The mean follow-up period after final restoration placement was 48.3 ± 14.4 months, ranging from 3 to 77 months. On average, each patient received 2.45 restorations, with a maximum of 12 restorations in one case.

Overall, 196 out of 209 restorations (90.6%) were successful, performed in 77 patients, while 13 restorations (9.4%) failed, occurring in 8 patients.

The primary cause of failure was ceramic fracture (*n* = 7), accounting for 70% of all failed cases, followed by chipping (*n* = 3, 30%).

The mean patient age was 180.98 months (approximately 15 years), with a mean follow-up period of 48.3 months and an average of 2.45 restored teeth per patient. Detailed descriptive statistics for age, follow-up period, and number of restored teeth are provided in Supplementary Table S1.

Table 1 summarizes the comparative analysis between feldspathic veneers and lithium disilicate crowns. Lithium disilicate crowns were used for a significantly higher number of teeth per patient (*p* = 0.015), while no significant differences were observed in follow-up duration or patient age between the two groups.

There was no significant association between the type of ceramic restoration and patient sex (Fisher’s Exact Test, *p* = 0.708), suggesting a balanced clinical indication for both materials across genders.

A significant association was found between the type of ceramic restoration and clinical outcome. All failures occurred in the feldspathic veneer group, while lithium disilicate crowns exhibited a 100% success rate (Fisher’s Exact Test, *p* < 0.001), as seen in Table 2.

No statistically significant associations were found between the clinical outcome (success vs. failure) and patient age, number of restored teeth, or follow-up duration. Patients with failed restorations had a slightly higher mean age (188.1 months vs. 180.2 months), fewer restored teeth (1.75 vs. 2.52), and a shorter follow-up period (40.3 vs. 49.1 months), but these differences were not statistically significant (Mann–Whitney U test; *p* > 0.05 for all comparisons).

These findings suggest that lithium disilicate crowns may offer superior long-term clinical outcomes compared to feldspathic veneers in anterior restorations, particularly in pediatric anterior trauma cases, where structural integrity is critical.

Regarding the distribution of restored teeth across the 85 patients included in the study, the most frequently treated tooth was the maxillary central incisor, which was restored in all cases (100%). The maxillary lateral incisors were restored in 14 patients (16.5%), followed by the maxillary canines in 5 patients (5.9%). Among the mandibular anterior teeth, central incisors were treated in six patients (7.1%), while only two patients (2.4%) received restorations on mandibular lateral incisors. The least commonly restored teeth were the mandibular canines, with a single patient (1.2%) presenting restorations in this region. These results reflect the predominance of maxillary central incisor trauma in pediatric anterior injuries and the relatively limited extension of trauma to other anterior units. Restoration patterns varied considerably between patients, underscoring the heterogeneous nature of anterior dental trauma in this population. No statistically significant associations were found between the type of restored tooth and the clinical outcome (*p* > 0.05 for all subgroups).

Detailed restoration patterns per patient further highlights this heterogeneity. One patient received full anterior rehabilitation involving all maxillary and mandibular incisors and canines, while another received restorations for all upper and lower incisors. Two patients had restorations covering maxillary central and lateral incisors as well as canines. Four cases involved both maxillary and mandibular central incisors. Six patients were treated with lithium disilicate crowns for maxillary central and lateral incisors. A single male patient presented with restorations exclusively on the maxillary lateral incisors due to deep overbite and palatal trauma. The most common clinical scenario involved bilateral restorations of the maxillary central incisors (32 patients), while six patients required restoration of only one central incisor. Among those treated with feldspathic veneers, 7 patients received a single veneer on a maxillary central incisor, 23 received veneers on both central incisors, and 2 patients underwent full anterior rehabilitation (canine to canine) with six feldspathic veneers.

In total, 144 lithium disilicate crowns and 65 feldspathic ceramic veneers were placed, amounting to 209 anterior restorations in 85 patients.

To further assess the durability of the two ceramic systems, a Kaplan–Meier survival analysis was conducted. The mean restricted survival time was significantly higher for lithium disilicate crowns (77 months), with no recorded failures during the follow-up period. In contrast, feldspathic veneers exhibited a restricted mean survival of 63.7 months, with 13 failures out of 65 restorations, as detailed in Table 3.

**Figure 3:**
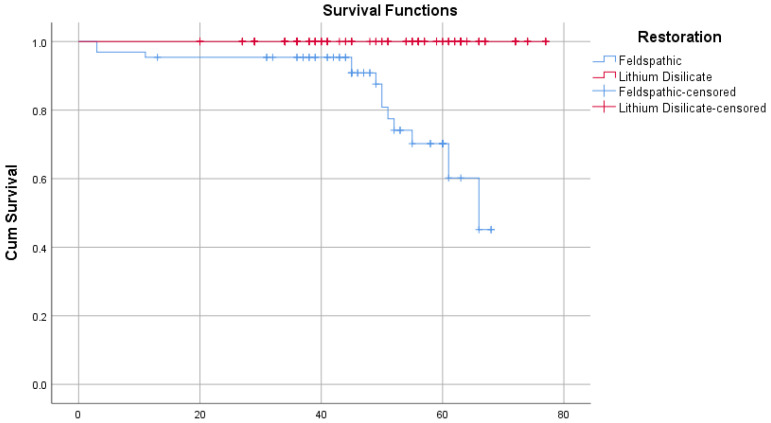
Kaplan–Meier survival curves showing superior longevity of lithium disilicate crowns compared to feldspathic veneers (Log-rank test, *p* < 0.001).

This difference was statistically significant (Log-rank test, χ^2^ = 40.735, *p* < 0.001), with the survival curves clearly separating over time, as shown in Figure 3.

Although restorations with favorable outcomes had a slightly longer mean follow-up duration (50.2 vs. 41.6 months), this difference was not statistically significant (Mann–Whitney U test, *p* = 0.302).

## 4. Discussion

The contemporary management of anterior dental trauma in adolescents must address not only biological and functional aspects but also significant psychosocial consequences. The appearance of anterior teeth is crucial to a child’s self-image, social interactions, and emotional development, particularly during adolescence, when sensitivity to physical appearance and peer perception is heightened [27,28]. Dental trauma, discoloration, and abnormalities in tooth shape or size can disrupt normal psychosocial development, leading to withdrawal, diminished self-esteem, and even long-term emotional distress [28]. As a result, minimally invasive restorative strategies have become a clinical priority in pediatric dentistry [29], with an emphasis on preserving healthy tooth structure while meeting both functional and esthetic demands. This reflects the ongoing shift toward conservative, patient-centered care.

The prevalence and distribution of dental trauma observed in this study closely mirror national data. A study from Târgu Mureș, Romania, reported similar findings regarding age, gender, and the predominance of anterior maxillary involvement, supporting the representativeness of our sample within the Romanian pediatric population [30]. Comparisons with international data from Nigeria, Iraq, Greece, Switzerland, and the Czech Republic reveal parallel trends, particularly the higher incidence in boys, frequent trauma to maxillary central incisors, and a predominance of enamel–dentin fractures caused by falls or sports-related activities [31,32,33,34,35]. These consistent patterns across populations enhance the external validity of our findings and highlight a global need for early, conservative intervention [30,31,32,33,34,35].

Although there is substantial literature that supports the clinical performance of all-ceramic restorations in adults, including follow-ups extending over a decade, there remains a notable gap in equivalent data for children [36,37,38,39,40]. Most existing evidence regarding the longevity of feldspathic porcelain and lithium disilicate restorations is derived from adult cohorts, which limits its extrapolation to pediatric cases [36,37]. Biological and functional differences between children and adults, such as tissue maturation and occlusal dynamics, may significantly influence restorative outcomes [38]. Without dedicated studies in younger populations, clinicians face uncertainty in selecting and forecasting the long-term performance of these materials in adolescents [39,40].

Recent advancements in pediatric dentistry have increasingly prioritized minimally invasive approaches that focus on tissue preservation, early detection, and tailored outcomes. Innovations, including bioactive materials, resin infiltration, laser therapy, and silver diamine fluoride, have broadened therapeutic possibilities [41,42]. Additionally, digital tools like three-dimensional printing and artificial intelligence are improving diagnostic accuracy and individualized treatment [41]. However, these approaches are often limited by the lack of pediatric-specific longitudinal data and require clinician expertise, careful case selection, and, frequently, a multidisciplinary framework, especially in trauma management [43]. In complex scenarios, collaboration between pediatric dentists, endodontists, orthodontists, prosthodontists, and oral and maxillofacial surgeons becomes essential for addressing both the immediate injury and its sequelae [43,44]. Despite promising results, long-term validation remains insufficient, as much of the literature draws on adult populations or early clinical outcomes [42,45]. Moreover, inconsistent integration into dental training programs further hampers uniform clinical adoption [46,47,48].

Children from socially vulnerable backgrounds are disproportionately affected by dental trauma. This is particularly true among children with autism spectrum disorders [49], those diagnosed with ADHD and treated pharmacologically [50], or those with neurodevelopmental conditions that increase the risk of falls or self-injury. In these high-risk groups, interdisciplinary collaboration is not merely beneficial but necessary for comprehensive diagnosis, monitoring, and care. Coordinated efforts involving pediatric dentists, orthodontists, prosthodontists, oral surgeons, and behavioral specialists are crucial in delivering outcomes that meet both functional and psychosocial needs [51]. In these contexts, conservative restorative approaches are often the standard of care rather than an option, as the severity and recurrence of trauma in these populations demand tailored, multidisciplinary planning. However, the literature still lacks robust long-term outcomes for these vulnerable groups [52]. Greater awareness of the link between dental trauma and quality of life is needed to inform both clinical and public health strategies [49,50,51,52].

**Figure 4:**
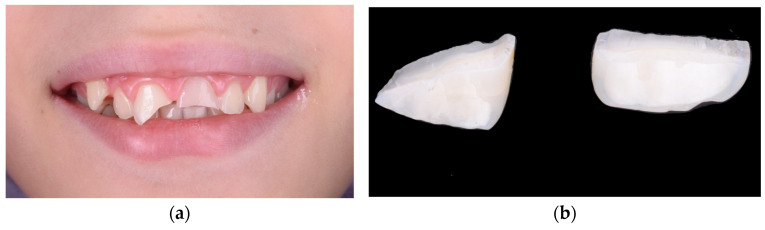

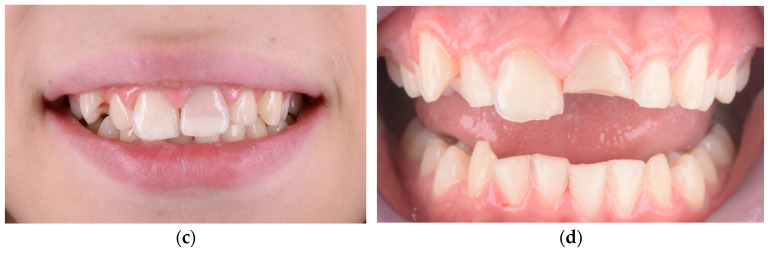
(**a**) Initial presentation showing crown fractures affecting both maxillary central incisors. (**b**) The two detached fragments retrieved intact, with well-defined fracture lines allowing for possible reattachment. (**c**) Immediate clinical appearance following fragment reattachment, showing acceptable alignment, although a noticeable color mismatch remains between the tooth structure and the reattached fragments. (**d**) Failure of both reattached fragments over time, with visible debonding and incisal-edge fractures under functional load.

**Figure 5:**
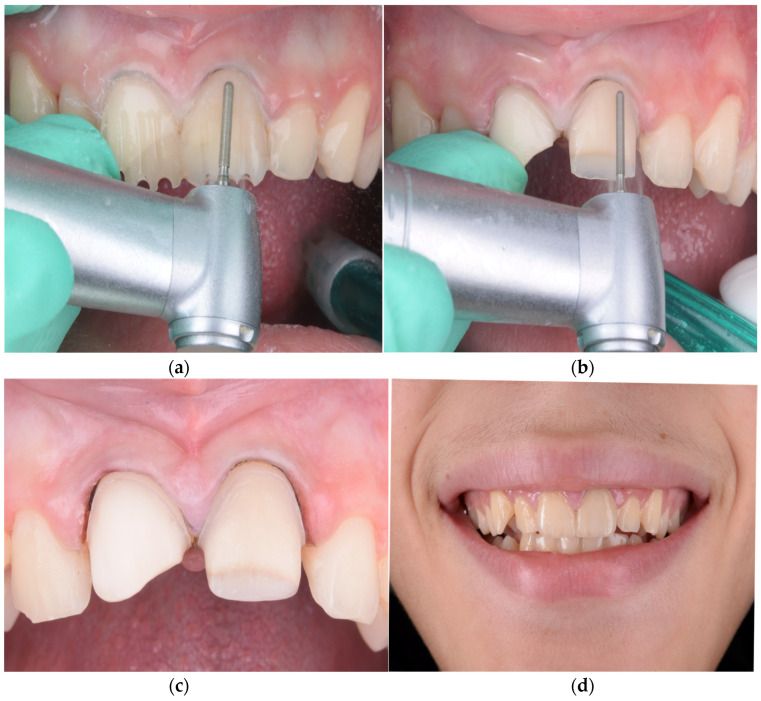
(**a**) Guided tooth preparation using the mock-up for precise reduction and preservation of enamel. (**b**) During preparation, the color mismatch between the natural tooth and the previously reattached fragment became more evident. (**c**) Final aspect of the prepared teeth immediately before cementation of the lithium disilicate restorations. (**d**) Esthetic outcome following adhesive placement of lithium disilicate crowns, illustrating morphology consistent with the adjacent teeth and shade matching the surrounding dentition.

In the initial cases of this study, we employed fragment reattachment, a conservative technique frequently used in pediatric dental trauma [53,54,55]. However, our clinical outcomes indicated poor long-term stability and noticeable color discrepancies between the tooth and the reattached fragment, prompting us to discontinue this technique for the remainder of the study. The procedural steps and limitations of one such case are illustrated in Figure 4 and Figure 5. After the detachment or fracture of the reattached fragments, the affected teeth were subsequently restored using lithium disilicate crowns, which demonstrated superior esthetic and mechanical performance throughout the observation period.

There is a clear lack of long-term clinical data on the use of minimally invasive prosthetic restorations in pediatric anterior teeth, particularly those made from all-ceramic materials such as lithium disilicate and feldspathic porcelain.

**Figure 6:**
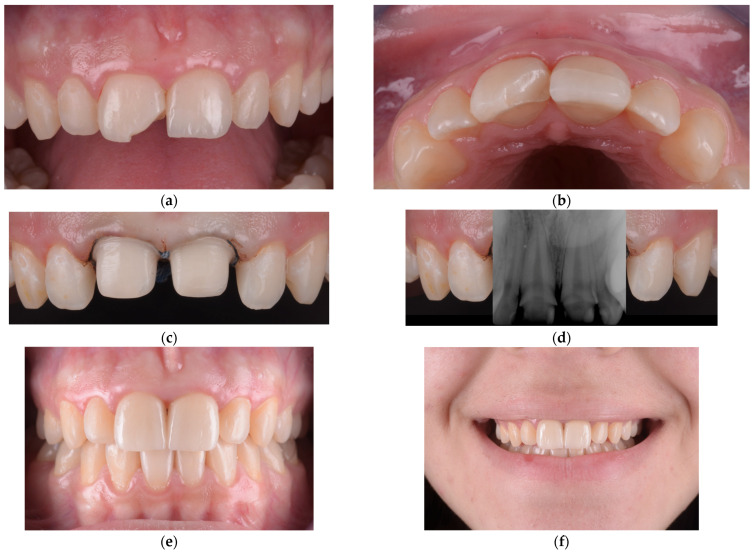
(**a**) Fracture of the feldspathic ceramic veneer affecting the incisal third of the maxillary left central incisor. (**b**) Occlusal view confirming the extent and morphology of the ceramic fracture. (**c**) Tooth preparations for lithium disilicate restorations, with retraction cords placed to avoid biological width violation. (**d**) Periapical radiograph illustrating the large pulp chamber volume and confirming that vitality was preserved following conservative preparation. (**e**) Intraoral frontal view of the lithium disilicate crowns after final cementation. (**f**) One-month recall showing a smile with color and morphology matching the adjacent teeth, demonstrating stable esthetic integration within the dental arch.

In our clinical experience, some feldspathic ceramic veneers failed over time, primarily due to chipping or fracture under functional load. In such cases, the affected teeth were prepared for lithium disilicate restorations, which offer superior mechanical resistance and better long-term performance in structurally compromised teeth. This approach mirrored our management of failed fragment reattachments, where lithium disilicate crowns were likewise used as a reliable restorative alternative. One such case is presented in Figure 6, illustrating the clinical workflow and the material adaptation following veneer failure.

The different preparation depths and retention forms of veneers compared to full crowns inevitably influence their long-term prognosis. While veneers preserve a higher proportion of sound tooth structure and benefit from enamel bonding, their thinner ceramic layers are more prone to superficial chipping. Conversely, full crowns provide greater structural reinforcement but at the cost of increased tooth reduction. These inherent differences must be considered when interpreting survival rates, as the material’s performance cannot be entirely separated from the restoration type.

Feldspathic ceramic veneers were deliberately chosen in this cohort because they allow for an extremely conservative or even no-preparation approach, preserving maximum enamel structure and pulp vitality—an essential consideration in adolescent patients. Moreover, feldspathic ceramics exhibit unparalleled optical properties, including high translucency and natural fluorescence, which are critical for achieving seamless integration in the anterior esthetic zone. While newer materials such as lithium disilicate and monolithic zirconia provide enhanced strength, they typically require greater tooth reduction and compromise the minimally invasive philosophy that guided the treatment of these young patients. Therefore, feldspathic veneers represented the most appropriate option at the time, aligning with both the esthetic demands and the biological considerations specific to this age group.

Future studies should focus on evaluating the longevity, esthetic stability, and biological integration of these restorations in children, considering their specific functional and developmental context. Comparative trials and interdisciplinary treatment protocols are also needed to support evidence-based decision making in complex trauma cases.

## 5. Conclusions

In pediatric dentistry, managing anterior dental trauma requires restorative strategies that combine esthetic outcomes with long-term durability. In this study, lithium disilicate crowns showed a 100% success rate over a follow-up period of up to 60 months, making them highly predictable in structurally compromised teeth. Feldspathic veneers also performed well, with an 86.2% success rate, although most failures involved chipping under functional load. In such cases, the failed veneers were successfully replaced with lithium disilicate restorations, which provided improved resistance and esthetic stability. These findings support the selective use of both ceramic systems, with lithium disilicate being preferable in cases involving greater tissue loss or functional stress. Moreover, optimal outcomes were achieved through close multidisciplinary collaboration.

## Figures and Tables

**Table 1:** Comparative analysis between feldspathic veneers and lithium disilicate crowns with respect to number of restorations per patient, follow-up duration, and patient age.

Variable	Feldspathic (*n* = 32)	Lithium Disilicate (*n* = 53)	*p*-Value
Number of teeth restored	2.00 ± 1.14	2.72 ± 1.90	0.015
Follow-up duration (months)	44.72 ± 15.07	50.43 ± 13.68	0.166
Patient age (months)	183.34 ± 21.74	179.55 ± 23.72	0.519

**Table 2:** Distribution of clinical outcomes (success and failure) for feldspathic veneers and lithium disilicate crowns. Values are expressed as number of restorations, with the percentage within each outcome category shown in parentheses.

Outcome	Feldspathic	Lithium Disilicate	Total
Failure	8 (100.0%)	0 (0.0%)	8 (100.0%)
Success	24 (31.2%)	53 (68.8%)	77 (100.0%)
Total	32 (37.6%)	53 (62.4%)	85 (100.0%)

**Table 3:** Association between restoration type and clinical outcome (success/failure).

Kaplan–Meier Summary Table							
						95% CI	
Restoration	N	Fail	Restricted Mean	Standard Error	Median Survival	Lower	Upper
Feldspathic	65	13	63.69	3.008	66	61	69.7
Lithium Disilicate	144	0	77	0			

## Data Availability

The original contributions presented in this study are included in the article/Supplementary Materials.

## Supplementary Materials

The following supporting information can be downloaded at https://www.mdpi.com/article/10.3390/children12081019/s1. Table S1: Descriptive statistics for patient age, follow-up period, and number of restored teeth.
