# Supplementary material for: Long-Term Performance of Feldspathic and Lithium Disilicate Restorations in Pediatric Anterior Dental Trauma
Source: Children (Basel). 2025 Aug 1;12(8):1019. doi: 10.3390/children12081019 (PMC12384468; doi:10.3390/children12081019)
Supplement: Supplementary file 1 [file children-12-01019-s001.zip › children-3741525-supplementary.pdf]

Table S1. Descriptive statistics for patient age, follow-up period, and number of restored teeth.

Descriptive Statistics (n=85)	Age (months)	Time Since Placement (months)	Number of Restored Teeth
Mean	180.98	48.28	2.45
Std. Deviation	22.94	14.4	1.68
Minimum	133.0	3.0	1.0
Maximum	215.0	77.0	12.0
Median	186.0	49.0	2.0
1st Quartile	158.0	39.0	2.0
3rd Quartile	199.0	60.0	2.0
